# The machining torch movement for the rectangular plasma sheet metal cut

**DOI:** 10.1371/journal.pone.0291184

**Published:** 2023-09-14

**Authors:** Alvaro Neuenfeldt Júnior, Matheus Francescatto, Olinto Araújo, David Disconzi, Gabriel Stieler

**Affiliations:** 1 Innovation and Competitiveness Group, Production Engineering Post-Graduation Program, Federal University of Santa Maria, Santa Maria, Brazil; 2 Production Engineering Post-Graduation Program, Industrial Technical College of Santa Maria, Santa Maria, Brazil; National Taiwan University of Science and Technology, TAIWAN

## Abstract

The sheet metals can be cut into rectangular or irregular pieces, where the main objective is to minimize the sheet metal’s physical waste. However, the operating time, the number of movements to cut all rectangles edges, and the cutting process quality, must be considered. The objective of this research was to compare the machining torch movement behavior using optimal and alternative solutions for rectangular plasma sheet metal cuts. A bottom-left-fill heuristic and a tabu search heuristic were used to find the alternative solution, while the optimal solution was obtained with a mixed-integer linear programming. The comparison was developed considering three parameters: the total processing time, the effective distance traveled by the machining torch to cut the metal, and the movement distance traveled by the machining torch without cutting the metal. The packing layout given by alternative solutions can reduce the operational cutting processing time and the distance covered by the machining torch movement. From an economic perspective, optimal solutions are recommended when compared to alternative solutions given the lower expenses with raw material waste.

## Introduction

In the industrial context, manufacturing efficiency is verified when the quantity and quality of manufactured products with zero defects are obtained, reducing operational costs, using rationally raw materials and inputs, as well as the available operational time [1, 2]. The metal-mechanical sector is a key part of the steel production chain, which is based on iron and mineral commodities, being both scarce resources and with complex extraction processes.

In metal-mechanical manufacturing processes, sheet metals are cut into rectangular or irregular pieces to be used as components for the assembly of main structures in a final product, being scientifically characterized as a cutting and packing problem. Furthermore, the problem is considered an open-dimensional problem in which a Cartesian coordinate system is used as a reference for the cutting plane of rectangular items, also called the rectangular two-dimensional strip packing problem (2D-SPP). The main objective is to minimize the height (or length) or maximize the area used to pack all small items (rectangles) in the strip (sheet metal), without overlapping [3–6]. A packing layout can be found manually, using algorithms based on heuristics [7–11] to obtain good quality alternative solutions or exact methods [12–14] to find the optimal solution with optimality proved.

For an industrial scale, the sheet metal cutting process is generally performed using cutting machining with a computer numeric control table. The cutting process with a computer numeric control table is given by the torch movement, based on the plasma cutting machine characteristics, input data, and rectangles’ packing layout on the sheet metal. In plasma cutting, an ionized gas (e.g., oxygen, nitrogen, or argon-hydrogen) at high temperature is injected through a torch with a cutting nozzle used to compress the ionized gas [15]. As a common cutting process in the metal-mechanical sector, plasma cutting machines are competitive for speed, average cutting precision, and relatively low purchase value. On the opposite, factors such as dimensional tolerance allowed and rectangles’ finishing quality are listed as the main disadvantages [16, 17].

Considering the direct relation between the cutting process and the problem instances’ packing layout, the sheet metal height minimization is the objective function used to obtain an optimal solution in the classic version of the rectangular 2D-SPP. However, an extensive version demands the study of additional aspects of sheet metal physical waste, such as the operational cutting processing time related to the number of movements needed for the machining torch to cut all rectangles edges and complete the cutting process with quality.

The objective of this research was to compare the machining torch movement behavior using optimal and alternative solutions for rectangular plasma sheet metal cuts. Furthermore, the main contributions are: (1) Develop a methodological research framework to evaluate a practical aspect related to rectangular plasma sheet metal cut, which can be used as a reference for similar research in other cutting and packing problems; (2) Verify if the alternative solutions are capable of reducing the operational cutting processing time and the distance covered by the machining torch movement during the plasma cutting process when compared to the optimal solutions obtained using the sheet metal physical waste minimization as objective function; (3) Contribute to improving the manufacturing efficiency in the metal-mechanical sector, specifically for the rectangular sheet metal cutting process, verifying the impact in the raw material waste and the operational cutting processing time, mainly for a practical demand found in the production of steel and aluminum small rectangles welded to assemble trucks’ bodywork.

This paper is structured as follows: Section 2 presents the research framework with the defined technical and operational parameters. Section 3 presents the results obtained with the comparison proposed and aspects related to the machining torch movement behavior. Finally, Section 4 presents some final remarks and future research ideas.

## Research framework

**Fig 1:**
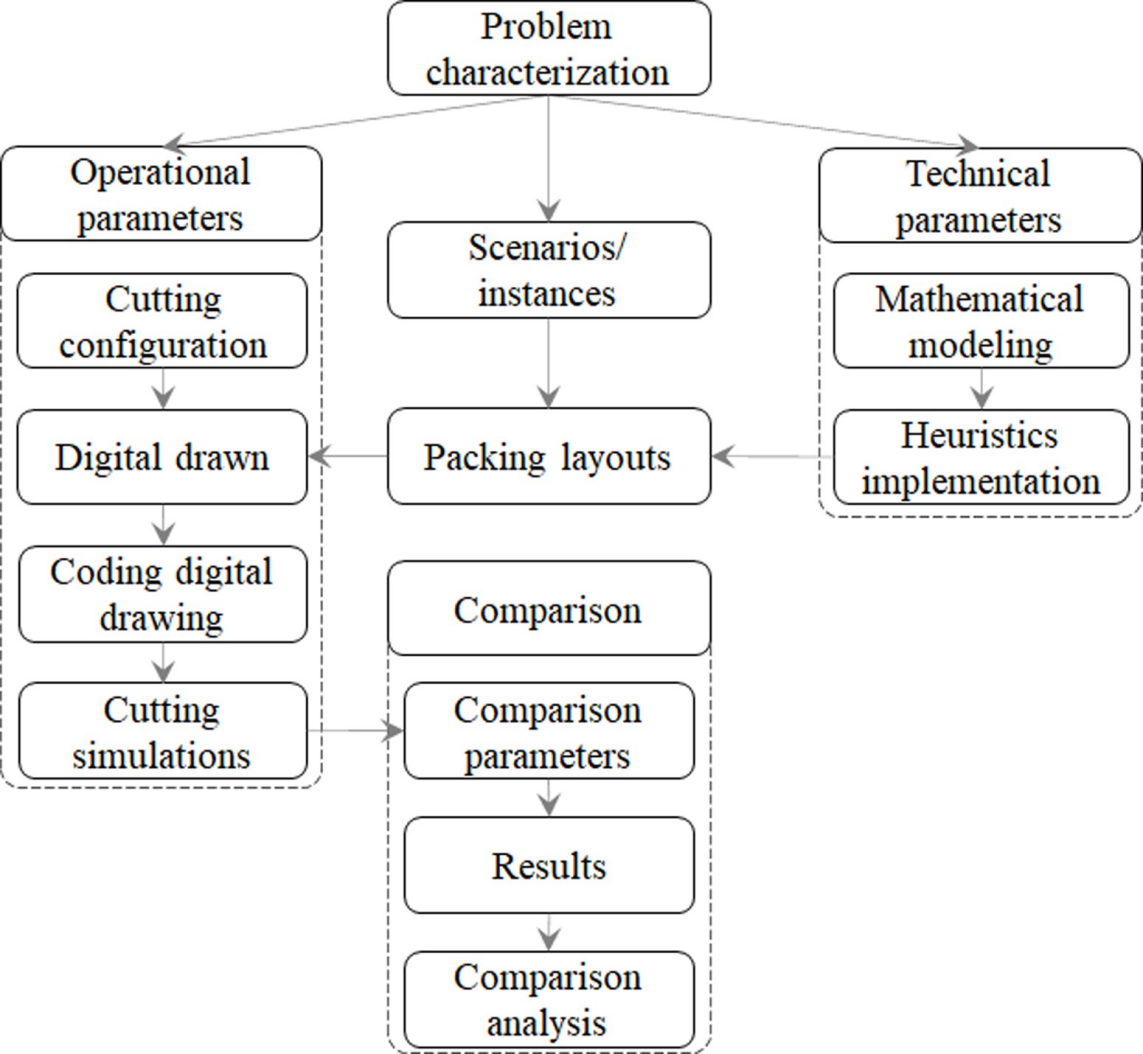
Research framework.

**Fig 2:**
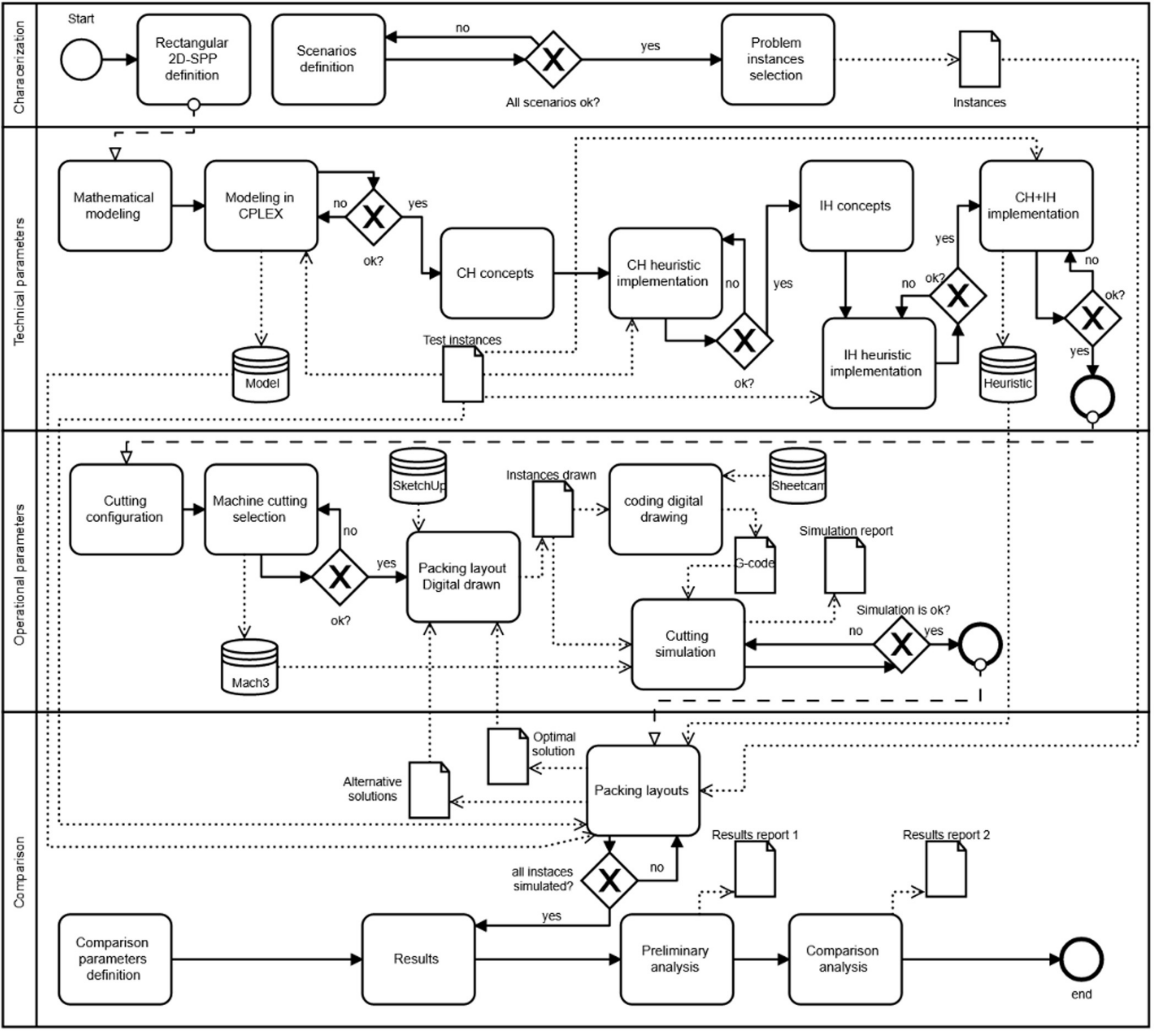
Business process model and notation research framework.

The research framework, shown in Fig 1, was conducted in structured steps according to all input data necessary to obtain results reports as outputs, enabling descriptive and technical analyzes throughout the plasma sheet metal cutting process. The research framework was developed to verify the machining torch movement behavior in four main steps: Problem characterization; rectangular 2D-SPP technical parameters; cutting machine operational parameters; and comparison. Details about each step are shown in the next sections (see the research framework in the business process model in Fig 2 and notation format in Table 1).

**Table 1:** Basic notation.

Notation	Type of notation	Description
*I*	Dataset	Set of rectangles
*i*	Index	Rectangle belonging to *I*
*h*_*i*_	Input data (Model, Algorithm)	Rectangle’s height
*w*_*i*_	Input data (Model, Algorithm)	Rectangle’s width
*x*_*i*_	Decision variable (Model)	Rectangle’s position in the *x*-axis
*y*_*i*_	Decision variable (Model)	Rectangle’s position in the *y*-axis
*α*_*i*_	Decision variable (Model)	Boolean variable
*z*_1*ij*_	Decision variable (Model)	Binary variable to define if the rectangle is allocated at the left of a positioned rectangle
*z*_2*ij*_	Decision variable (Model)	Binary variable to define if the rectangle is allocated at the right of a positioned rectangle
*Z*_3*ij*_	Decision variable (Model)	Binary variable to define if the rectangle is allocated below a positioned rectangle
*Z*_4*ij*_	Decision variable (Model)	Binary variable to define if the rectangle is allocated above a positioned rectangle
*pi*	Index	Problem instance index
*W* ^*pi*^	Input data (Model, Algorithm)	Strip’s width
*n* ^*pi*^	Input data (Model, Algorithm)	Number of rectangles
*nt* ^*pi*^	Input data (Algorithm)	Number of different rectangles
$D_{1}^{pi}$	Output data (Algorithm)	Higher strip’s dimension
$D_{2}^{pi}$	Output data (Algorithm)	Lower strip’s dimension
$d_{1i}^{pi}$	Output data (Algorithm)	Higher rectangle’s dimension
$d_{2i}^{pi}$	Output data (Algorithm)	Lower rectangle’s dimension
*ar* ^*pi*^	Output data (Comparison)	Aspectratio
*ht* ^*pi*^	Output data (Comparison)	Heterogeneity
*p*	Index	Complete and feasible solutions search space
$H_{p}^{pi}$	Output data (Algorithm)	Strip’s height solution from search space (*p*)
$H_{OS}^{pi}$	Output data (Model)	Strip’s height solution from the optimal solution
$H_{A1}^{pi}$	Output data (Algorithm)	Strip’s height solution from alternative solution 1
$H_{A2}^{pi}$	Output data (Algorithm)	Strip’s height solution from alternative solution 2
*px*_1_	Input data (Algorithm)	Empty strip space *x*-axis coordinate
*py*_1_	Input data (Algorithm)	Empty strip space *y*-axis coordinate
*LB* ^*pi*^	Input data (Algorithm)	Area lower bound from Martello et al. [12]
$Ct_{p}^{pi}$	Output data (Comparison)	Comparison parameters “Cut”
$Dp_{p}^{pi}$	Output data (Comparison)	Comparison parameters “Displace”
$Tm_{p}^{pi}$	Output data (Comparison)	Comparison parameters “Time”

For the characterization step, the rectangular 2D-SPP conditions delimitations were proposed, based on the cutting characteristics found in industrial practice to verify the machining torch movement. To organize the comparison analysis, simulation scenarios were defined to relate the results obtained with the variation of rectangular 2D-SPP characterization variables, from [18], with the machining torch movement behavior during the cutting process. Problem instances from the literature are adopted considering the above and below mean characterization variables values for each simulation scenario. Next, technical parameters (Section 2.1) were developed through an exact method modeling and a constructive and improvement heuristic algorithm implementation. Test instances were used to verify if all the rectangular 2D-SPP conditions were correctly considered. As outputs, optimal solutions and alternative solutions for problem instances are obtained using, respectively, the mathematical model given by the exact modeling and the heuristic algorithm.

Operational parameters (Section 2.2) initiate with the cutting configuration based on physicochemical aspects from rectangles and sheet metal characteristics, followed by the selection of a machine capable of providing a computational solution to simulate the cutting process. The packing layouts from problem instances must be digitally drawn using, for example, the SketchUp® 2020 (version 2020-0-1), encoding in computer numeric control, G-code, and the rectangles’ coordinates into the strip with the Sheetcam® (version 6.0.0) to feed the Mach3® simulation software. In the end, a report is generated and the simulation is finished. This procedure is standard and adopted to simulate any type of problem instance. The comparison step (Section 3) is conducted using the problem instances’ packing layouts from the optimal and alternative solutions defined for each scenario. Finally, the results obtained with the simulation are compiled in preliminary and comparison analysis, generating two outputs, results report 1 and 2, enabling the discussion about the machining torch movement behavior. In this study, the computer’s processor is an Intel® Core™ i5-7200U 2.5GHz, with 8 GB of RAM and a Windows 10 64-bit operating system.

### Technical parameters

**Fig 3:**
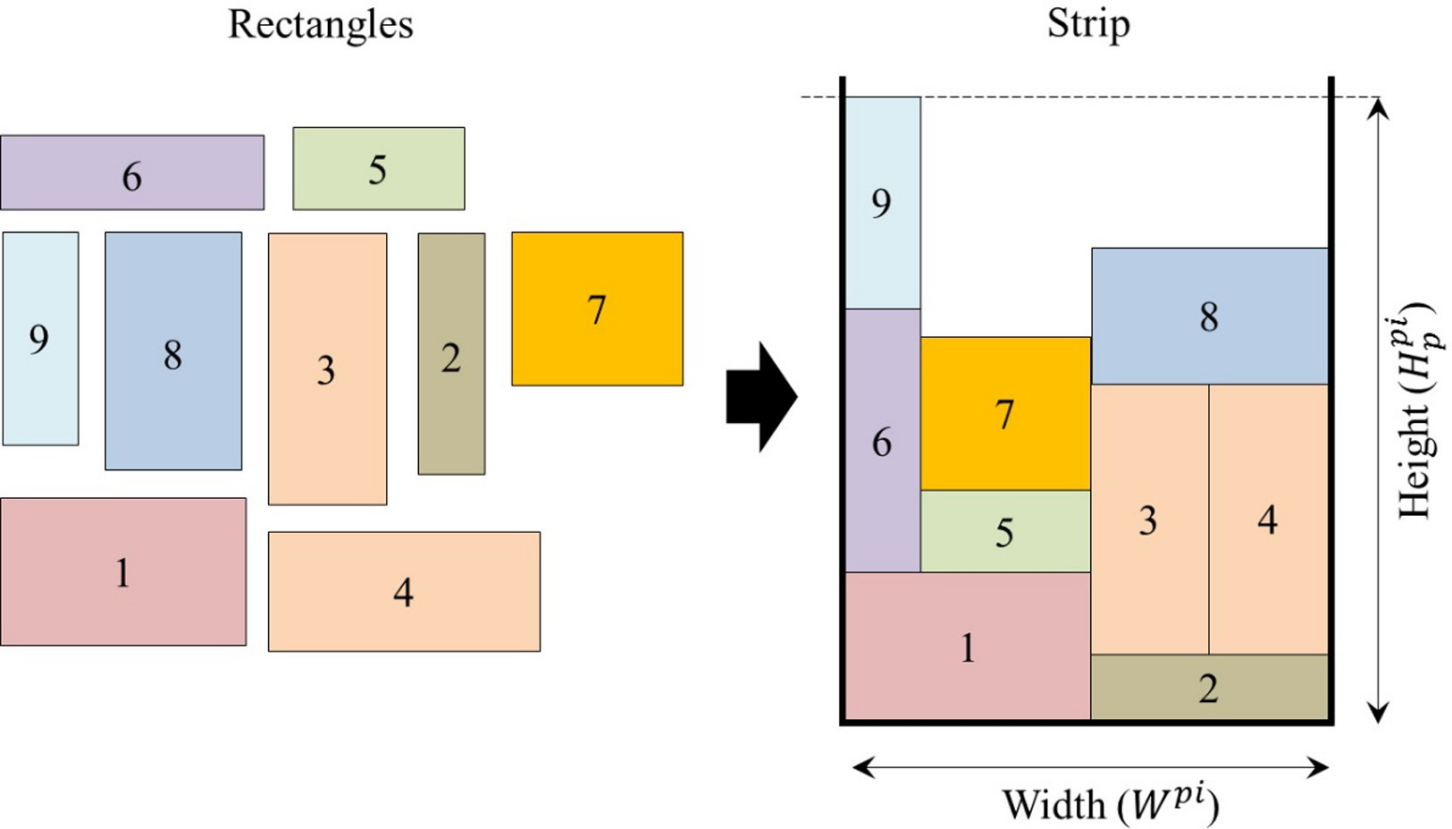
Rectangular 2D-SPP.

For the plasma sheet metal cutting process using a machine with a computer numeric control table, represented by the rectangular 2D-SPP, the width (*W*^*pi*^) of the strip (sheet metal) is finite and predefined, and the second dimension, the height or length ($H_{p}^{pi}$), is considered infinite and must be calculated based on all rectangles’ packing layouts. Thus, the objective is to minimize the infinite dimension, reducing the sheet metal physical wastes, as shown in Fig 3. The 90° rotations are allowed, respecting orthogonality constraints. Furthermore, the input data is offline, where parameters such as width, area, rectangles’ perimeter, and the strip width are known in advance, enabling the input sequence preorder in which the rectangles are packed.

#### Related works

To find a problem instance strip height, solution methods were developed over the years in favor of the search for efficient solutions, being widely classified as exact, heuristics, approximation, and hybrid methods [19, 20]. For each problem instance, packing layouts are obtained using exact methods and heuristics. In specific for the last years, [21] developed two algorithms to solve the multiple strip packing problem, where each rectangle’s width and height is dependent on the strip available to be packed. The first algorithm is a skyline best-fit heuristic combined with a randomized local search, and the second algorithm distributes the rectangles among the strips with an integer program, posteriorly packing the rectangles with a best-fit heuristic. Also, the approach was effective in instances adapted from the literature and large-scale synthetically generated problems related to task scheduling. A deep reinforcement learning algorithm to solve the 2D-SPP was proposed in [22], where a model-free reinforcement learning algorithm trains network parameters to optimize the packing sequence, using deep networks with self-learning characteristics to solve different instances. Furthermore, an algorithm is designed to determine rectangles positions and calculate model rewards and packing parameters. The experimental results show the proposed algorithm can produce better or comparable results when compared with classical heuristics algorithms.

A hybrid metaheuristic approach that relies on a local search algorithm to deliver satisfying rectangles placements on the horizontal axis and an exact procedure to find rectangles positions on the vertical axis is shown in [23] for the 2D-SPP, being competitive on moderate-sized instances when compared to the best-known approaches. Also, [24] used the positions and covering methodology to obtain exact solutions for the 2D-SPP, generating a set of valid positions in which a rectangle can be packed into the strip and, with a set-covering formulation, selecting the best configuration of rectangles. Also, the method was found to be useful for small and medium-size instances, as well as proving optimality for new literature instances. Finally, [25] presented two improved metaheuristics for the 2D-SPP. The first metaheuristic is a hybrid approach combining simulated annealing with a heuristic construction algorithm, and the second metaheuristic applies simulated annealing directly in completely define layouts. Furthermore, in some instances, the algorithms outperform existing strip packing metaheuristics.

Exact methods are developed through mathematical modeling to determine the best resource use, given the objectives and needs of the problem. In [12] a new relaxation for the rectangular 2D-SPP is shown, which produces good results based on a branch-and-bound algorithm, allowing to solve some problem instances with up to 200 rectangles. In [26], a new branch-and-bound algorithm was developed from a search tree structure based on [13] to try new limits and conditions to improve the algorithm’s efficiency. Kenmochi et al. [13] proposed exact algorithms for the rectangular 2D-SPP with and without 90° rotations. A branch-and-bound algorithm was developed, examining two operation points based on the strip lower left point and on a strategy called G-Staircase, together with new delimitation operations based on dynamic programming and linear programming. To improve the results found in [13], in [27], a new branch-and-bound strategy adding only canonical forms of solutions during the iteration process was successfully proposed.

#### Mixed-integer linear programming model

Additionally, Table 1 introduces the basic notation used in this research for mathematical model, constructive and improvement heuristics algorithm, and comparison analysis.

Considering the mathematical models proposed in [12, 19, 26], and mainly in [13], the mathematical model adopted considers the strip variables $H_{OS}^{pi}$ and *W*^*pi*^ as integer values, to pack a set of *I* = {1, 2,…,*n*} previously known rectangles with dimensions *w*_*i*_ and *h*_*i*_, being possible to define the input sequence order and position, *x*_*i*_ and *y*_*i*_, of each rectangle on the strip. The orthogonality constraint is guaranteed with the use of a Boolean variable *α*_*i*_, which contains two values, true or false, represented by 1 and 0, respectively. If the rectangle does not rotate, *α*_*i*_ = 1 and (1 ‒ *α*_*i*_) = 0, maintaining the rectangles’ original dimensions. However, if the rectangle is rotated, then *α*_*i*_ = 0 and (1 ‒ *α*_*i*_) = 1, changing *h*_*i*_ to *w*_*i*_ and vice versa. Thus, the numerical set of rectangles is doubled where the height value *h*_*i*_ can also be read as a width value *w*_*i*_, allowing 90° rotations. The rectangles packing follows a bottom-left orientation, using the coordinates *x*_*i*_ = 0 and *y*_*i*_ = 0 as the origin.

The objective function (Eq 1) is related to $H_{OS}^{pi}$ minimization. Constraints (2) refers to the rectangles positioning inside the strip, meaning the coordinate *x*_*i*_ added to *w*_*i*_ or *h*_*i*_ cannot exceed *W*^*pi*^. Similarly, in constraints (3), the coordinate *y*_*i*_ added to *h*_*i*_ or *w*_*i*_ cannot exceed $H_{OS}^{pi}$.

$$\text{minimize}\;H_{OS}^{pi}$$ (1)

$$\text{subject}\;\text{to}\;x_{i}+w_{i}\alpha_{i}+h_{i}\left(1-\alpha_{i}\right)\leq W^{pi}\;\;\forall i\in I,$$ (2)

$$y_{i}+h_{i}\alpha_{i}+w_{i}\left(1-\alpha_{i}\right)\leq H_{OS}^{pi}\;\forall i\in I,$$ (3)

$$x_{i}+h_{i}\left(1-\alpha_{i}\right)+w_{i}\alpha_{i}\leq x_{j}+M\left(1-z_{1ij}\right)\;\forall i,j\in I,i\neq j,$$ (4)

$$x_{j}+h_{j}\left(1-\alpha_{j}\right)+w_{j}\alpha_{j}\leq x_{i}+M\left(1-z_{2ij}\right)\;\forall i,j\in I,i\neq j,$$ (5)

$$y_{i}+h_{i}\alpha_{i}+w_{i}\left(1-\alpha_{i}\right)\leq y_{j}+M\left(1-z_{3ij}\right)\;\forall i,j\in I,i\neq j,$$ (6)

$$\begin{array}{cc} & y_{j}+h_{j}\alpha_{j}+w_{j}\left(1-\alpha_{j}\right)\leq y_{i}+M\left(1-z_{4ij}\right)\;\forall i,j\in I,i\neq j, \end{array}$$ (7)

$$z_{1ij}+z_{2ij}+z_{3ij}+z_{4ij}=1\;\;\forall i,j\in I,i\neq j,$$ (8)

$$x_{i},y_{i}\geq 0\;\forall i\in I,$$ (9)

$$\alpha_{i},z_{1ij},z_{2ij},z_{3ij},z_{4ij}\in \{0,1\}\;\forall i,j\in I.$$ (10)

Constraints (4), (5), (6), and (7) ensure that there is no overlap between rectangles by using the Big-M method. Also, *z*_1*ij*_,*z*_2*ij*_,*z*_3*ij*_, and *z*_4*ij*_ are binary decision variables used with the Big-M method to avoid rectangle overlapping, where *z*_1*ij*_ = 1determines if the rectangle is allocated at the left of a rectangle already positioned, *z*_2*ij*_ = 1 determines if the rectangle is allocated at the right of a rectangle already positioned, *z*_3*ij*_ = 1 determines if the rectangle is allocated below a rectangle already positioned, and *z*_4*ij*_ = 1 determines if the rectangle is allocated above a rectangle already positioned. Thus, during the packing process, the rectangles coordinates, *x*_*i*_ and *y*_*i*_, added to the corresponding rectangle dimension, *w*_*i*_ or *h*_*i*_, are considered to define the final rectangle position. Constraints (8) guarantees that only one constraint between (4), (5), (6), and (7) is valid for any two rectangles. Constraints (9) is the non-negativity constraint. Finally, constraints (10) characterizes the values which *α*_*i*_,*z*_1*ij*_,*z*_2*ij*_,*z*_3*ij*_,*z*_4*ij*_ can assume. To obtain the optimal solution of a problem instance, the mathematical modeling was developed through mixed-integer linear programming (MILP) and implemented on IBM ILOG CPLEX Optimization Studio 12.5–64 bits.

#### Constructive and improvement heuristics algorithm

Heuristics allow an improvement in the efficiency of search solutions, without guaranteeing the solution optimality [19]. In the constructive heuristics, the execution is incremental, where at each loop a rectangle is positioned in the strip until reach a complete solution. For the Bottom-Left-Fill (BLF), each rectangle is packed in the lowest and left feasible position in the strip, with the possibility of occupying empty spaces between packed rectangles [28]. Improvement heuristics are used to improve the quality of an initial solution generated randomly or by a constructive heuristic, exploring the feasible solutions space [29]. Tabu search is an adaptive procedure to guide the local search in the continuous exploration of the search space, exploring the immediacy of a current solution, even if the solution quality is worse compared to solutions already found, allowing the escape from local optima [30]. The information from previous iterations is memorized through a set of short-term and long-term prohibitions lists. At each iteration, prohibitions are introduced to not allow changes of some rectangles in the sequence order.

In this research, alternative solutions are obtained using the BLF constructive heuristic, together with an improvement process using the tabu search. Both BLF and tabu search were implemented in C/C++, following the logic presented in Algorithm 1. In the initialization (lines 1 to 7), the search for two alternative solutions starts with the preset of the rectangular 2D-SPP input parameters, strip and rectangles characteristics (lines 1 to 4), short-term and long-term prohibitions lists, TS parameters, and the maximum number of iterations (lines 5 and 6), and BLF parameters, in specific the construction packing procedures (line 7). For the procedure (lines 8 to 34), at each iteration (line 8), the TS parameters are reset and the rectangles’ input sequence order is randomly modified (lines 9 and 10), keeping unchanged the input position of rectangles penalized in both short-term and long-term prohibitions lists. Next, each rectangle (line 11) is individually packed in the lower and left empty spaces available in the strip, according to the rectangular 2D-SPP constraints (lines 15 to 23). The Boolean variable *c1* is used to validate the relation between the rectangle’s dimensions and the first empty space available. If the relation between the dimensions and the empty space is feasible, without overlapping with other rectangles already allocated and without overlapping the object’s boundaries, the rectangle can be allocated in the empty space and then *c1* is equal to 1. Also, the strip space used is eliminated and new empty strip spaces are set considering the layout with the new rectangle allocated. After packing all rectangles (lines 24 to 29), the rectangles’ positions *x*_*i*_ and *y*_*i*_ and the $H_{p}^{pi}$ are reported, the short-term and long-term list penalties are randomly applied after defining the next input sequence order, excluding rectangles with the input positions already penalized. With all iterations processed, the packing layout from two alternative solutions ($H_{A1}^{pi}$ and $H_{A2}^{pi}$) are randomly selected and the heuristic is finished (lines 30 to 34).

For problem instances with up to 100 rectangles, when selected for the short-term list, the rectangle input position cannot be moved in the next eight consecutive iterations. If selected for the long-term list, the rectangle input position cannot be moved in the next 15 consecutive iterations. The penalties decrease unitarily to zero. The maximum number of iterations (*maxit*) was set at 200, regardless of the problem instance size. Also, alternative solutions from exact methods can be obtained using as stopping criterion a predefined computational processing time. However, the implemented heuristic was used to control the solutions quality from alternative solutions. To perform comparative analysis, the ideal is to obtain not similar alternative solutions with different packing layouts from the optimal solution.

### Operational parameters

Developed approximately forty years ago, the plasma cutting process is often used to cut fine or thick metal surfaces (up to 25 mm for mild steel, 20 mm for stainless steel, and 16 mm for aluminum), using an active plasma arc cutting based on a high-velocity ionized gas to melt a localized sheet metal surface. The cutting is proceeded in steps, beginning with the arc ignition. Once the pilot arc is created, the gas flow blows the pilot arc out of the torch nozzle creating an arc loop protruding. With the sheet metal positioned, the power supply active an arc transfer and the nozzle is removed from the circuit, becomes an anode, and the main current establishes between the nozzle and the sheet metal. The plasma cutting process is given by the torch movement until all rectangles’ edges are completely separated from the rest of the sheet metal [31]. More details about the plasma cutting process technical characteristics using a machine with a computer numeric control table can be found in [32–34].

## Algorithm 1 — Pseudocode for the implemented heuristic

Initialization
1. **set** strip width *0* = {*W*^*pi*^};
2. **set** empty strip spaces coordinates *Px* = {*px*_1_, *px*_2_, …, *px*_*m*_} and *Py* = {*py*_1_, *py*_2_, …, *py*_*m*_};
3. **set** rectangles *I* = {1, 2, …, *n*};
4. **set** rectangles dimensions *Ix* = {*w*_1_, *w*_2_, …, *w*_*n*_} and *Iy* = {*h*_1_, *h*_2_, …, *h*_*n*_};
5. **set** shortlist, longlist, and TS parameters;
6. **set** max number of iterations *maxit*;
7. **set** BLF parameters;
Procedure
8. **for** each (TS iteration) **do**
9. **reset** TS parameters;
10. **set** rectangles input sequence order;
11. **for** each (rectangle) do
12.  **reset** BLF parameters;
13.  **read** rectangles dimensions;
14.  **set** empty strip spaces decreasing order;
15.  **for** each (empty strip space) **do**
16.  **set** a Boolean validation variable *c1*;
17.  **while** (*c1* = 0) **do**
18.   **read** empty strip spaces coordinates;
19.   **pack** rectangle;
20.   **if** (rectangle packing = feasible) **then**
21.   *c1* = 1;
22.   **eliminate** used strip space;
23.   **set** new empty strip spaces;
24.  **if** (all rectangles packed) **then**
25.   **stop** BLF process;
26.   **report** rectangles positions;
27.   **report** $H_{p}^{pi},D_{1}^{pi},D_{2}^{pi},d_{1i}^{pi},d_{2i}^{pi};$
28.   **set** shortlist penalty;
29.   **set** longlist penalty;
30. **if** (TS iteration = *maxit*) **then**
31.  **select** alternative solution 1 $H_{A1}^{pi}$;
32.  **select** alternative solution 2 $H_{A2}^{pi}$;
33.  **stop** procedure;
34. **end**.

The torch movement control in the *x* and *y* axes is automatic using a computer numeric control table, operated with minimum requirements which must be developed before the manufacturing process, such as: Torch cooled by the coolant; packing layout must not exceed the strip dimensions; electrical ground; and keep within the upper and lower limits the sheet metal thickness [35].

The operational parameters are based on physicochemical aspects and the rectangles’ characteristics required. Considering as reference the Hypertherm® Powermax45 XP® cutting machine, a total of nine operational parameters were described, as shown in Table 2. As references, each operational parameter value was defined based on the machine cutting operator manual [15], in experimental tests, or with the practical experience of machine operators consulted to conduct the study, mainly for values not declared in the machine cutting operator manual.

**Table 2:** Operational parameters.

Operational parameter	Unit of measure	Value	Reference
Sheet thickness	mm	6	Practical experience
Rectangles’ cutting distance	mm	0	Experimental tests
Kerf width	mm	0.02	[15]
Preheat time	seconds	2	Experimental tests
Feed rate	mm/min	1000	[15]
Pierce delay	seconds	1	[15]
Pierce height	mm	5	[15]
Plunge rate	mm/min	5	Practical experience
Cut height	mm	2	[15]

The “rectangles’ cutting distance” is used to indicate the distance between edges of neighbor rectangles, which can change significantly the packing layout according to the distance adopted, especially if the sheet metal width is relatively similar to the rectangle dimensions. The “kerf width” is used to scale the torch nozzle diameter, influencing the active plasma arc size and the “rectangles’ cutting distance”, where a larger arc requires a minimum distance between edges to avoid cutting neighbor rectangles with wrong dimensions. The “preheat time” is necessary to heat the components before starting the cutting process. Although, the “preheat time” to plasma machine components is practically minimal, especially for fine sheet metal surfaces.

The “feed rate” is the speed when the active plasma arc is operational. The higher the “feed rate”, the less time the active arc is in contact with the region of the sheet metal being cut, which is not feasible for thick metal surfaces or hard types of metals, similar to verified for “pierce delay”, “cut height”, and “cut height”. The “pierce delay” is the time when the active arc plasma stays static to drill vertically the sheet metal. The “pierce height” is relative to the distance between the torch nozzle and the sheet metal surface before the active arc is operational, and the “cut height” is the distance between the torch nozzle and the sheet metal surface when the active arc is operational. If the torch nozzle is located with a short “cut height” concerning the sheet metal surface, after cutting a rectangle, the sheet metal can release and shift vertically upwards, which causes a collision with the torch nozzle, resulting in failures in the next rectangles cuts. Finally, in the “plunge rate”, the torch nozzle descent speed from the “pierce height” to the “cut height” is set.

Being the cut trajectory pre-defined based on the torch movement algorithm developed in the Mach3® simulation software, the “cutting rule” is the command used to define the cutting sequence logic, where three options are available: “all inside first”; “shortest path”; and “keep parts together”. In the “all inside first”, the first cut is developed in all rectangles with internal holes (if any), and in a second moment, the edges cut are proceeded. In the “shortest path”, the shortest distance covered to cut all rectangles is prioritized. In the “keep parts together”, all inside holes and edges of one rectangle are completely cut before proceeding to the next rectangles. In this study, the “shortest path” was selected as the “cutting rule”, where the plasma cutting process starts at the origin coordinate (*x*_*i*_ = 0 and *y*_*i*_ = 0), located at the lower and left positions in the sheet metal. To complement the operational parameters data, the ionized gas used is oxygen, being efficient, easy to operate, and relatively simple to find at an affordable cost. The type of sheet metal material is AISI 1020 mild carbon steel.

## Comparison

### Comparison parameters

Physical or non-physical wastes are non-add value factors in product manufacturing. For the plasma sheet metal cutting process, factors such as the operational cutting processing time required to cut all rectangles’ edges, the quantity of sheet metal unused, inconsistent definition of sheet metal thickness not following the products’ technical characteristics, poor finishing quality in disagreement with technical as, for example, ISO 9013:2002 [36], energy inefficiency, incorrect cutting execution speed, and machining torch movements can be used to quantify physical or non-physical wastes.

The comparison parameters to verify the machining torch movement behavior for the plasma sheet metal cutting process are expressed in two non-add value variables, “Time” ($Tm_{p}^{pi}$) and “Displace” ($Dp_{p}^{pi}$), and one add value variable, “Cut” ($Ct_{p}^{pi}$). The “Time” (in seconds) is the continuous numerical parameter measured from the first machining torch movement since the origin coordinates *x*_*i*_ = 0 and *y*_*i*_ = 0 until the end of the last rectangle edge cut, which can be positioned at any sheet metal coordinate (*x*_*i*_, *y*_*i*_). The movement distance (in millimeters), called “Displace”, is defined by the machining torch movement not used to cut some of the rectangles’ edges, found when the start of the next edge cut is not coincident with the end of the previous edge cut or when the first edge cut is not located in the origin coordinates *x*_*i*_ = 0 and *y*_*i*_ = 0, and must be reduced as much as possible. The last variable is the added value cutting distance (in millimeters), called “Cut”, which is the entire movement traveled by the torch in which the sheet metal is effectively cut by the active plasma arc. As the raw material is manufactured and transformed into a product, the cutting distance adds value to the cutting process.

For each problem instance, the machining torch movement behavior comparison is possible by analyzing the rectangles’ packing layout between the optimal solution obtained with the MILP model and the two alternative solutions found using constructive (BLF) and improvement heuristics (TS). Regardless of the number of times where a specific packing layout of any problem instance is simulated, the machining torch movement behavior and the results for the three comparison parameters will be the same if the operational parameters are constant for all simulations.

### Scenarios

With the comparison parameters and the packing layouts for the optimal solution and alternative solutions, simulation scenarios were developed to compare the machining torch movement behavior, based on rectangular 2D-SPP aspects described using characterization variables such as the aspectratio, heterogeneity, number of rectangles, and strip width.

The aspectratio (*ar*^*pi*^) measures variations between rectangles’ dimensions [18]. A bigger *ar*^*pi*^ is observed when the interval between the minimum and maximum rectangles’ dimensions is wide [37]. The aspectratio is obtained through geometric characteristics as the largest ($D_{1}^{pi}$) and smallest ($D_{2}^{pi}$) strip dimensions, considered as a reference *W*^*pi*^ and the calculated area lower bound *LB*^*pi*^ [12], the largest ($d_{1i}^{pi}$) and smallest ($d_{2i}^{pi}$) dimension of each rectangle, and the total number of rectangles (*n*^*pi*^), as shown in Eq 11. The heterogeneity (*ht*^*pi*^) refers to the diversification of the rectangles’ geometric proportions, comparing the absolute dimensions, the number of different rectangles in the problem instance (*nt*^*pi*^), and *n*^*pi*^, as shown in Eq 12.

$$ar^{pi}=\left(D_{1}^{pi}/D_{2}^{pi}\right)/\left[\overset{n}{\underset{i=1}{\sum }}\left(d_{1i}^{pi}/d_{2i}^{pi}\right)/n^{pi}\right],\;\forall i\in I$$ (11)

$$ht^{pi}=nt^{pi}/n^{pi}$$ (12)

The aspects *n*^*pi*^ and strip width (*W*^*pi*^) were chosen as basic characteristics related to how to effectively solve rectangular 2D-SPP problem instances. Both *ar*^*pi*^ and *ht*^*pi*^ are characterization variables based on complex characteristics used to verify how a single problem instance can be diverse in the rectangles and strip geometries, which is easily used to define its level of complexity, especially when reasonable amounts of empty spaces are allowed in the optimal packing layout. Thus, scenarios are established with the four characterization variables’ average values, using as a reference 790 benchmark problem instances with a maximum of 50 rectangles and *W*^*pi*^ ≤ 500*mm* from the scientific literature datasets *C* [38], *N* [7], *cx* [39], *gcut* [40], *cgcut* [41], *ngcut* [42], *bwmv* [43, 44], *beng* [45], *nice/path* [37], and *pt* [46]. Therefore, the mean characterization variables values used as a reference for the scenarios are *ht*^*pi*^ = 20, *W*^*pi*^ = 200 *mm*, *ht*^*pi*^ = 0.6, and *ar*^*pi*^ = 1.0. A total of 16 scenarios were formed from all possibilities, considering the above and below values of the mean characterization variables found, as shown in Table 3.

**Table 3:** Scenarios definition.

Scenario	*ar* ^*pi*^	*ht* ^*pi*^	*n* ^*pi*^	*W* ^*pi*^	Scenario	*ar* ^*pi*^	*ht* ^*pi*^	*n* ^*pi*^	*W* ^*pi*^
1	≥1.0	≥0.6	≥20	≥200	9	≥1.0	≥0.6	<20	≥200
2	<1.0	≥0.6	≥20	≥200	10	<1.0	≥0.6	<20	≥200
3	≥1.0	<0.6	≥20	≥200	11	≥1.0	<0.6	<20	≥200
4	<1.0	<0.6	≥20	≥200	12	<1.0	<0.6	<20	≥200
5	≥1.0	≥0.6	≥20	<200	13	≥1.0	≥0.6	<20	<200
6	<1.0	≥0.6	≥20	<200	14	<1.0	≥0.6	<20	<200
7	≥1.0	<0.6	≥20	<200	15	≥1.0	<0.6	<20	<200
8	<1.0	<0.6	≥20	<200	16	<1.0	<0.6	<20	<200

More complex scenarios are defined by values above the references in at least three characterization variables, such as scenarios 1, 2, 3, 5, and 9. Intermediate scenarios are defined by two values above the mean characterization variables (4, 6, 7, 10, 11, and 13). The remaining scenarios are considered less complex (8, 12, 14, 15, and 16). A total of 15 problem instances were found in the literature and can be adopted in each scenario: *N1* [7], *bwmv4*, *bwmv52*, *bwmv254* [43], *pt16_26_84*, *pt1_24_60*, *pt2_23_42*, *pt13_26_84*, *pt3_24_89*, *pt14_24_89b*, *pt1_23_3*, *pt14_24_89*, *pt7_23_100*, *pt6_23_95*, and *pt15_22_84* [46]. No problem instance reached the scenario 1 characterization variables conditions or the optimal solution’s packing layout was not found. Next, for each problem instance, the optimal solution ($H_{OS}^{pi}$) was obtained through the mathematical model presented in Section 2.1 and the two alternative solutions ($H_{A1}^{pi}$ and $H_{A2}^{pi}$) were obtained using the BLF positioning heuristic with the TS improvement heuristic. Table 4 shows the characterization variables values and the solution given by the strip height.

**Table 4:** Problem instances configuration.

Scenario	*pi*	*ar* ^*pi*^	*ht* ^*pi*^	*n* ^*pi*^	*W* ^*pi*^	* $H_{OS}^{pi}$ *	* $H_{A1}^{pi}$ *	* $H_{A2}^{pi}$ *
2	*bwmv254*	0.48	1.00	20	300	140	169	149
3	*pt1_24_60*	1.26	0.27	22	230	77	84	93
4	*pt2_23_42*	0.85	0.30	20	329	146	183	164
5	*bwmv4*	1.35	1.00	20	10	49	52	55
6	*bwmv52*	0.64	1.00	20	30	16	17	17
7	*pt16_26_84*	2.66	0.20	23	134	680	779	696
8	*pt13_26_84*	0.23	0.21	23	134	277	291	291
9	*pt3_24_89*	1.05	0.71	7	229	81	110	96
10	*pt14_24_89b*	0.83	0.71	7	229	237	386	240
11	*pt1_23_3*	2.46	0.36	14	224	29	59	39
12	*pt14_24_89*	0.11	0.57	7	229	237	284	271
13	*pt7_23_100*	1.40	0.70	10	94	247	279	325
14	*N1*	0.48	0.90	10	40	40	60	44
15	*pt6_23_95*	3.90	0.44	16	69	1154	1173	1173
16	*pt15_22_84*	0.37	0.45	11	134	291	298	294

The optimal solutions and the alternative solutions found allowed to obtain different packing layouts for the rectangles’ packing in the strip. The maximum computation processing time used to find each solution was 600s. The optimality of the optimal solutions of all tested instances was proven. The alternative solutions of the problem instances *bwmv52*, *pt13_26_84*, and *pt6_23_95* have the same values, but the rectangles’ packing is different to compare the machining torch movement behavior.

### Comparison results

#### Main results

Although the optimal solutions minimize the number of empty spaces in the rectangles’ packing, only for four problem instances (*pt14_24_89b*, *pt7_23_100*, *pt14_24_89*, and *N1*) the “Time” to cut all rectangles with the optimal solution packing layout is lower compared to the alternative solutions packing layout (Table 5). This characteristic is similar to “Cut”, which shows a cause-effect trend between the trajectory defined to cut the sheet metal and the operational time required to complete all cutting process, confirmed by the Pearson correlation coefficient equal to 0.98 between “Time” and “Cut” values. The operational parameter rectangles’ cutting distance is equal to 0 mm and border cuts were not considered. Only a single cut is required to separate two adjacent rectangles. The “Cut” is efficient, but with real chances of losing finishing quality, given the highly active plasma arc heat, which can over-melt the sheet metal surface. To complement, the “Displace” for packing layouts from optimal solutions is higher in 14 of the 15 problem instances when compared to alternative solutions.

**Table 5:** Results obtained.

Scenario	*pi*	$H_{OS}^{pi}$			$H_{A1}^{pi}$			$H_{A2}^{pi}$		
		Time (*s*)	Cut (*mm*)	Displace (*mm*)	Time (*s*)	Cut (*mm*)	Displace (*mm*)	Time (*s*)	Cut (*mm*)	Displace (*mm*)
2	*bwmv254*	249	3172	2216	234	3090	1428	226	2989	1567
3	*pt1_24_60*	174	2094	1423	154	1899	1133	155	1920	1244
5	*bwmv4*	45	358	343	43	354	320	41	342	294
9	*pt3_24_89*	94	1220	564	91	1229	485	96	1277	704
4	*pt2_23_42*	255	3323	1897	246	3250	2085	241	3146	1811
6	*bwmv52*	50	416	299	43	367	266	42	356	310
7	*pt16_26_84*	262	3409	2157	251	3344	1682	268	3538	1847
10	*pt14_24_89b*	128	1774	607	139	1935	520	143	1974	672
11	*pt1_23_3*	96	1143	670	91	1083	855	91	1095	785
13	*pt7_23_100*	112	1485	739	122	1601	706	130	1808	752
8	*pt13_26_84*	213	2678	1695	190	2495	981	285	5096	3584
12	*pt14_24_89*	124	1712	688	132	1806	628	125	1699	750
14	*N1*	36	375	225	39	404	291	37	397	201
15	*pt6_23_95*	278	3739	2090	276	3736	1875	275	3735	1769
16	*pt15_22_84*	154	2084	694	133	1835	631	132	1808	445

The best values found for each problem instance are highlighted in green.

Regarding the simulation scenarios, for the most complex scenarios, the “Time” for packing layout from alternative solutions is better when compared with all optimal solutions. The effect of the rectangles’ geometry variability is observed in the “Time” results in behavior following the scenarios’ level of complexity. A comparison between the “Time” for alternative solutions packing layout with the optimal solutions showed an average advantage equal to 1% for optimal solutions when only the less complex scenario is considered. On the opposite, the “Time” difference is equal to -7% and -1% for more complex and intermediate scenarios, respectively. Less complex problem instances are easier to obtain optimal solutions with reduced computational processing times compared with complex problem instances, also reflected in the quality proximity of the optimal solution’s packing layouts compared to alternative solutions, where the average gap between the strip heights obtained with the best alternative solutions concerning the optimal solution for less complex problem instances is 6.4%, while for more complex problem instances, the average gap is greater than 10%. Thus, the practical usage of optimal solutions packing layouts using the strip height minimization as an objective function is attractive for less complex problem instances, even without considering the “Time” to modeling the rectangular 2D-SPP.

Regardless of the problem instances’ complexity, a deeper study on machining torch movement behavior concerning the rectangles’ packing in the strip was developed, specified in three comparison analysis topics to verify in detail different situations about the torch movement verified during this research, as well as some practical consequences for the plasma sheet metal cutting processes management.

#### First comparison topic

**Fig 4:**
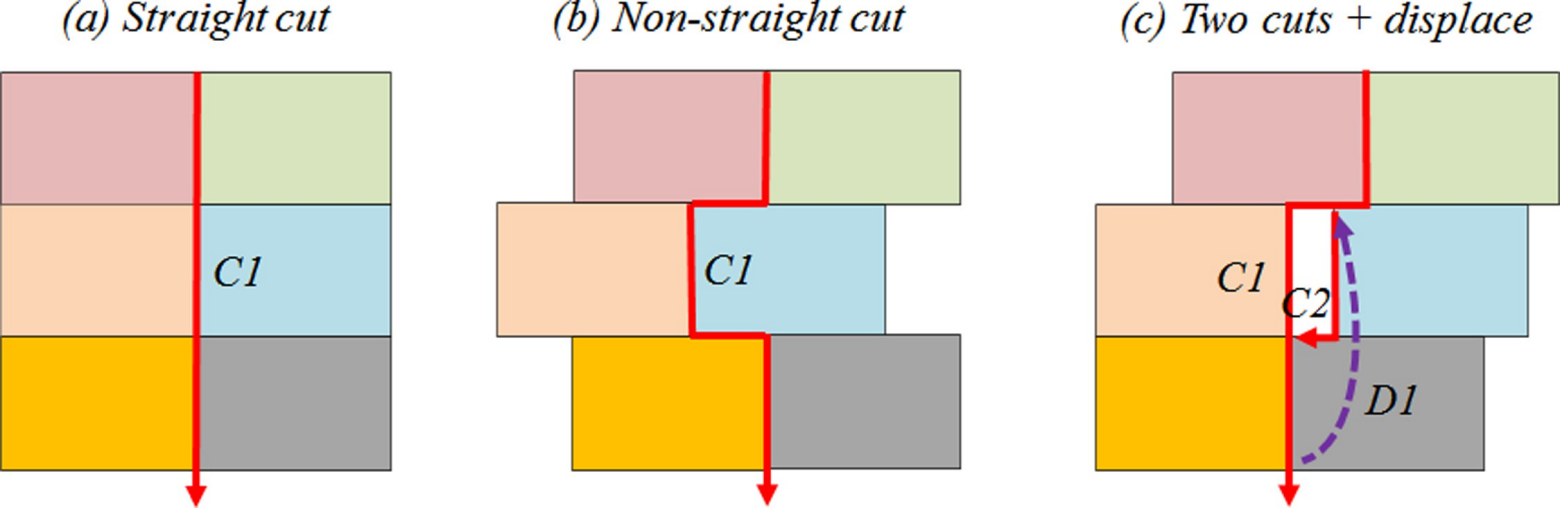
Examples of straight (a) and non-straight (b) cuts.

The first comparison topic analyzed is related to the packing layout, specifically the use of straight and non-straight cuts to minimize the strip height. Several cutting possibilities are allowed in the rectangular 2D-SPP, where straight-cut configuration allows cut movements with the shortest distances, which can be considered the most appropriate to reduce “Cut” movements. However, the non-straight movements are most common, given the irregular manner in which the rectangles are usually packed in the strip, especially for optimal solutions, where the use of available physical space must be maximum, regardless of whether similar rectangles are closely packed or not. Fig 4 shows three cut configurations with six standard rectangles. In the first configuration (Fig 4A) the straight cut is presented as the fastest possibility to separate all the rectangles in a single operation (C1), being the best option to reduce the operational cutting processing time wasted. In the second configuration (Fig 4B), the two intermediate rectangles were allocated in a non-straight manner, demanding more operational time to complete C1. Finally, a small physical waste between two rectangles is verified in Fig 4C, requiring an additional cut operation (C2) to finish the cutting process and a displacement movement to start C2, being the packing layout with a greater waste for both operational time and raw material.

The possibility of different cut configurations impacting the machining torch movement behavior was observed in the results obtained. For example, the strip height of alternative solutions 1 and 2 from problem instance *pt13_26_84* (*pi* = 8) is the same ($H_{A1}^{8}=H_{A2}^{8}=291$). However, the “Cut” and “Displace” movements behavior are completely different. In the alternative solution 2, “Cut” and “Displace” are $Ct_{A2}^{8}=5096\;mm$ and $Dp_{A2}^{8}=3584\;mm$, respectively. In the alternative solution 1, the comparison parameters are significantly lower, $Ct_{A1}^{8}=2495\;mm$ and $Dp_{A1}^{8}=981\;mm$, respectively. As a consequence, the physical waste with raw material is the same regardless of the alternative solution, but the operational cutting processing time required to cut all rectangles is 50% higher using the alternative solution 2, given the higher amount of non-straight cuts compared with the packing layout obtained in the alternative solution 1. From an economical point of view, considering the current industrial context, this movement waste causes a total cost 26% higher for alternative solution 2 compared with alternative solution 1 (Table 6), considering the cutting of only one sheet metal for a daily industrial workday.

**Table 6:** Raw-material waste and active plasma arc time to calculate the total financial costs for optimal and alternative solutions.

*pi*	$H_{OS}^{pi}$			* $H_{A1}^{pi}$ *			* $H_{A2}^{pi}$ *		
	Raw material waste (%)	Active arc time (s)	Total cost ($)	Raw material waste (%)	Active arc time (s)	Total cost ($)	Raw material waste (%)	Active arc time (s)	Total cost ($)
*pt16_26_84*	27.3	228	6.00	45.2	203	7.69	30.3	219	6.25
*pt6_23_95*	18.5	235	5.03	20.4	244	5.32	20.3	241	5.27
*pt14_24_89*	35.1	104	3.52	64.0	102	5.27	56.5	103	4.81
*bwmv254*	8.0	215	3.32	30.4	208	4.54	14.9	198	3.50
*pt2_23_42*	5.2	220	3.28	31.0	211	4.99	17.4	237	4.28
*pt14_24_89b*	21.5	119	3.02	46.6	127	4.85	23.0	131	3.29
*pt13_26_84*	6.7	201	2.96	13.3	175	2.99	13.3	229	3.78
*pt1_24_60*	10.5	144	2.18	20.5	128	2.21	33.4	131	2.56
*pt15_22_84*	6.3	138	2.17	8.8	122	2.08	7.4	121	1.99
*pt7_23_100*	6.4	99	1.52	20.2	108	2.10	38.7	114	2.79
*pt3_24_89*	10.5	82	1.34	50.1	81	2.36	31.0	85	1.91

The best values found for each problem instance are highlighted in green, the intermediate values in yellow, and the worst values in red.

For the problem instance *pt6_23_95* (*pi* = 15), the strip height is the same for both alternative solutions ($H_{A1}^{15}=H_{A2}^{15}=1173$). However, the rectangles’ packing layout is almost equal, reflecting similar “Cut” and “Displace” values, varying only 0.00026% and 0.0032%, respectively. In the problem instance pt15_22_84 (*pi* = 16), the calculated strip height is similar ($H_{OS}^{16}=291,H_{A1}^{16}=298$, and $H_{A2}^{16}=294$) and small changes in “Cut” and “Displace” behavior are verified, reflecting in a difference of 16% in the “Time” to execute the cuts between the “slower” packing layout, the optimal solution ($Tm_{OS}^{16}=154s$), compared to the packing layout with “faster” movement, verified for the alternative solution 2 ($Tm_{A2}^{16}=132s$), a consequence of the extensive demand for non-straight cuts to minimize as much as possible the strip height in the optimal solution’s packing layout.

**Fig 5:**
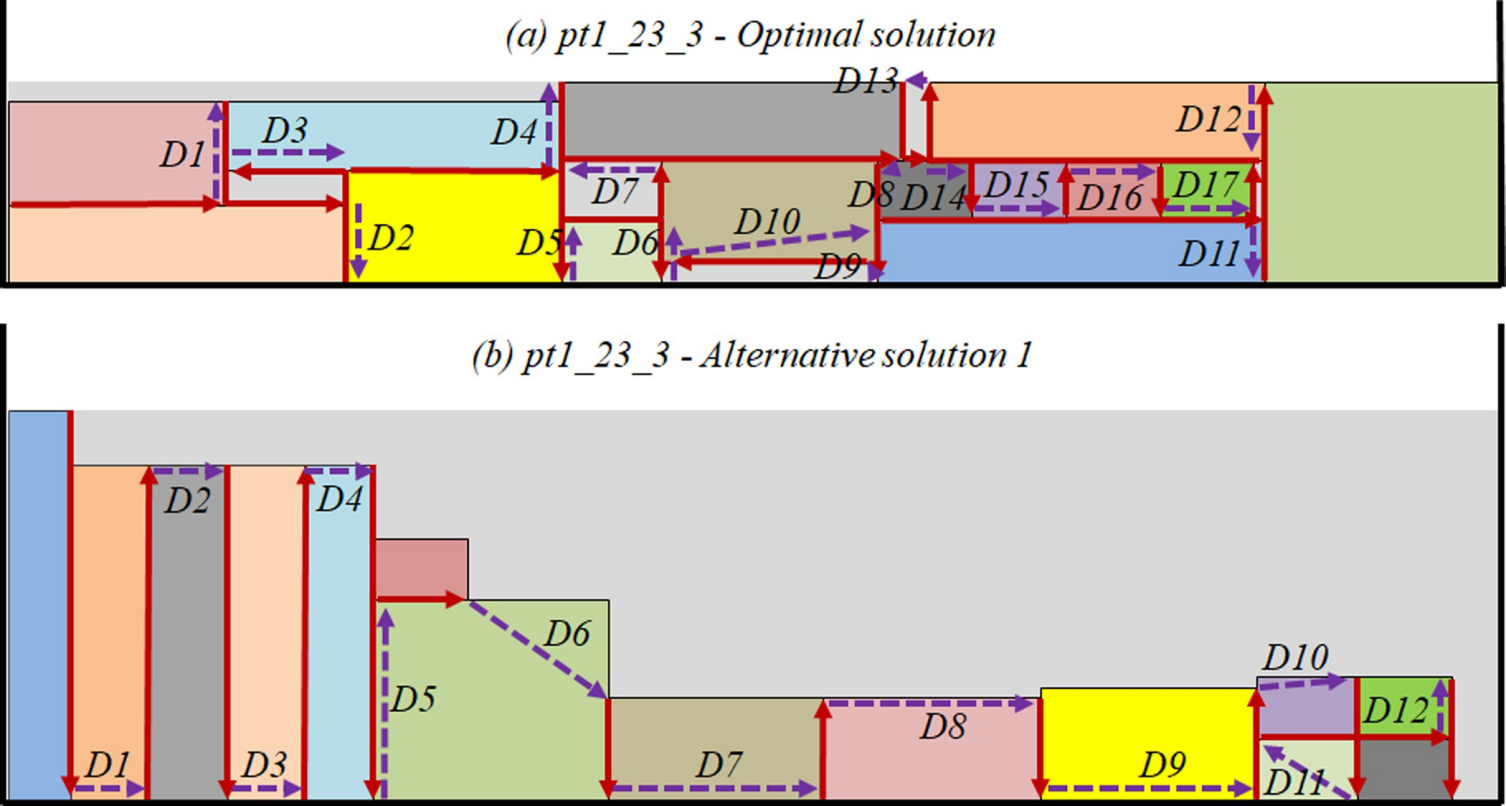
Cut movement of pt1_23_3 optimal (a) and alternative (b) solutions’ packing layouts.

To illustrate the impact of different packing layouts, Fig 5 shows two “Cut” movements of the problem instance *pt1_23_3*. For the optimal solution packing (Fig 5A), a total of 18 cuts must be performed to finish the cutting process, composed of short and long straight cuts, in addition to five non-straight cuts, resulting in a traveled distance of $Ct_{OS}^{11}=1143\;\;mm$. On the opposite, the alternative solution 1 packing layout (Fig 5B) allows the performance of only straight cuts, where the distance traveled by the cutting gun is $Ct_{A1}^{11}=1083\;\;mm$, 5.2% lower when compared to the optimal solution’s packing layout. Consequently, despite the “Displacement” of alternative solution 1 being 27.6% higher than the “Displacement” verified for the optimal solution, mainly given by the difference between the strip heights ($H_{OS}^{11}=29$ and $H_{A1}^{11}=59$), which forces the torch to travel longer distances, the “Time” required for the cutting process execution of alternative solution 1 is 5.2% lower when compared to the packing layout obtained with the optimal solution.

#### Second comparison topic

**Fig 6:**
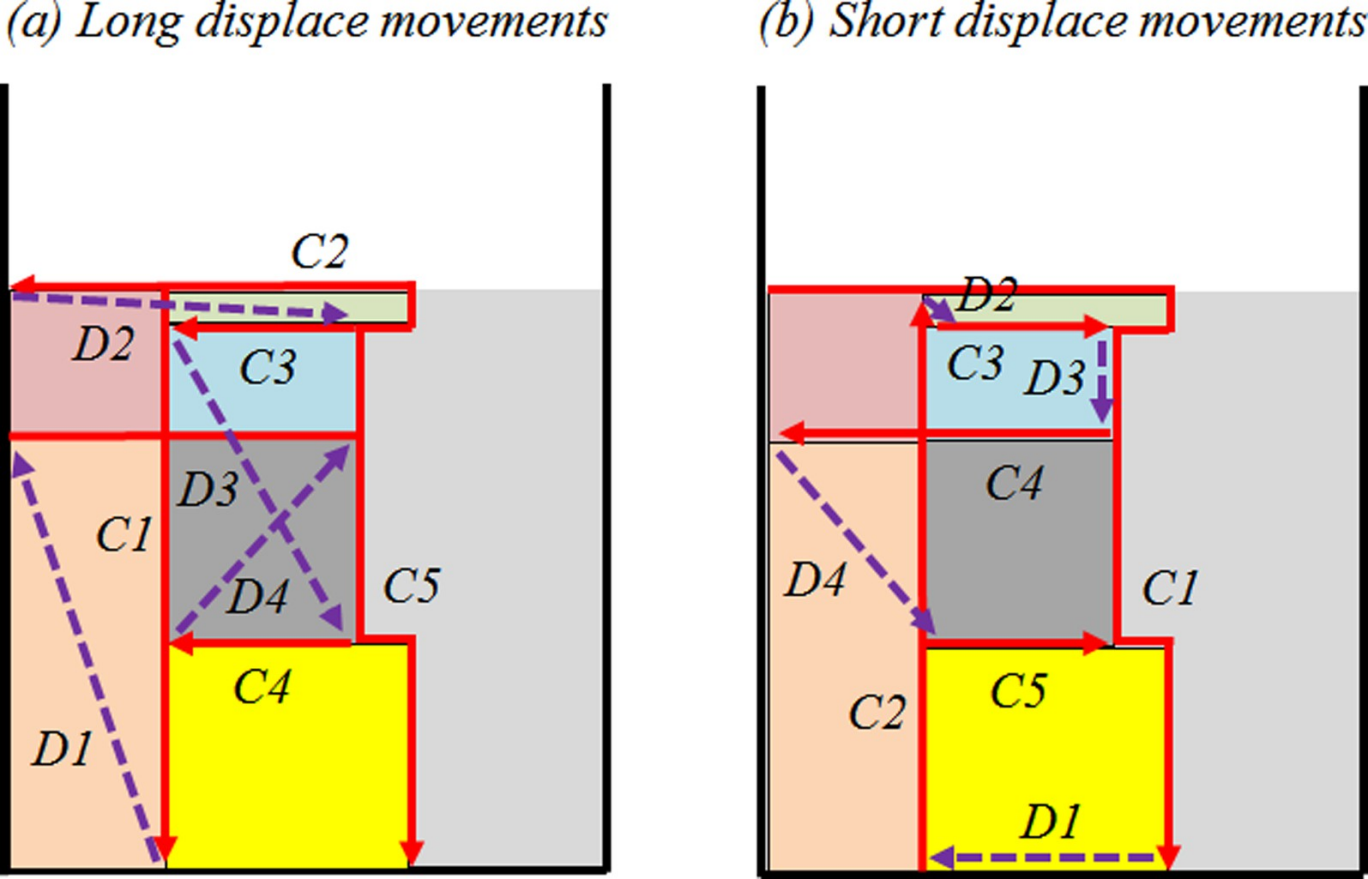
Examples of long (a) and short (b) displace movements.

The second comparison topic analyzed is related to torch displacement. Similar to straight and non-straight cuts for a single problem instance, equal or similar strip heights can be obtained using packing layouts with more displacement movements to start each rectangle cut. In addition, a packing layout can allow more than one machining torch movement behavior for both “Cut” and, mainly, for “Displace”. The example in Fig 6 shows a packing layout with two different torch movements. In Fig 6A, to perform five “Cuts” (C1–C5), a total of four (D1–D4) long “Displace” movements are required, while in Fig 6B, to cut the same rectangles, only three short “Displace” movements are required, reducing the operational cutting processing time needed to develop the entire cutting process, keeping the raw material waste constant.

Considering all problem instances tested, a direct relation between “Displacement” and “Cut” distances covered was not verified. In only seven problem instances (*pt1_24_60*, *bwmv4*, *pt2_23_42*, *pt16_26_84*, *pt13_26_84*, *pt6_23_95*, and *pt15_22_84*) the lowest “Displace” values are coincident with the lowest “Cut” movement values, despite both movements’ behavior performance being strongly related to the rectangles’ packing in the strip. In practice, for the cutting machine used in the simulation, the “Displace” velocity is an intrinsic parameter and cannot be changed, being higher when compared with the “Cut” velocity (ratio 1:5). Thus, greater distances traveled to start each “Cut” increase the operational cutting processing time required to cut all rectangles, being the comparison parameter “Time” more sensitive to variations in the distance covered for “Cut” concerning “Displace” movements. For example, in the problem instance *pt14_24_89* (*pi* = 12), similar “Displace” movements with different “Cut” values are verified. For the optimal solution, the “Time” required to cut all rectangles is 6% lower ($Tm_{OS}^{12}=124s$) compared to the alternative solution 1 ($Tm_{A1}^{12}=132s$), coinciding with the behavior of the results presented for “Cut” movement ($Ct_{OS}^{12}=1712\;mm$ and $Ct_{A1}^{12}=1806\;mm$), despite the “Displace” for the optimal solution being 9.5% higher than the alternative solution 1.

For the problem instance *pt14_24_89*, the smallest “Cut” movement was verified for alternative solution 2 ($Ct_{OS}^{12}=1699\;mm$). Despite the “Displace” movement being 9% higher compared to the optimal solution, the computational processing time is only one second higher, which shows the difference between “Cut” and “Displace” movements behavior in the computational processing time. Finally, the total financial cost using the packing layout provided by the optimal solution ($H_{OS}^{12}=237$) compared with the alternative solution 1 ($H_{A1}^{12}=284$) for a daily industrial workday to cut only one sheet metal is equal to $1.75 (see Table 5). Both “Displace” and “Cut” movements are defined based on the “cutting rule” input parameter, where the “shortest path” option was selected over “all inside first” and “keep parts together” options.

In the cutting process economic context, the “Displace” cost is lower than the “Cut” and raw-material physical waste costs. On average, the “Displace” cost is less than 1% of the “Cut” movement cost. For example, in the problem instance *N1*, using the measurement unit in millimeters as a reference and the optimal solution packing layout, the “Cut” cost to proceed with a single sheet metal is equal to $0.59 and the “Displace” movements cost is lower than $0.01. For this reason, “Displace” movements should not be prioritized when minimizing costs in the sheet metal cutting process with similar characteristics to those verified in this research. However, regarding the machining torch movement behavior, the study of “Displace” remains relevant to verify the rectangles’ packing layout efficiency in the sheet, mainly if the packing layout of similar rectangles is a practical requirement to be adopted.

#### Third comparison topic

**Fig 7:**
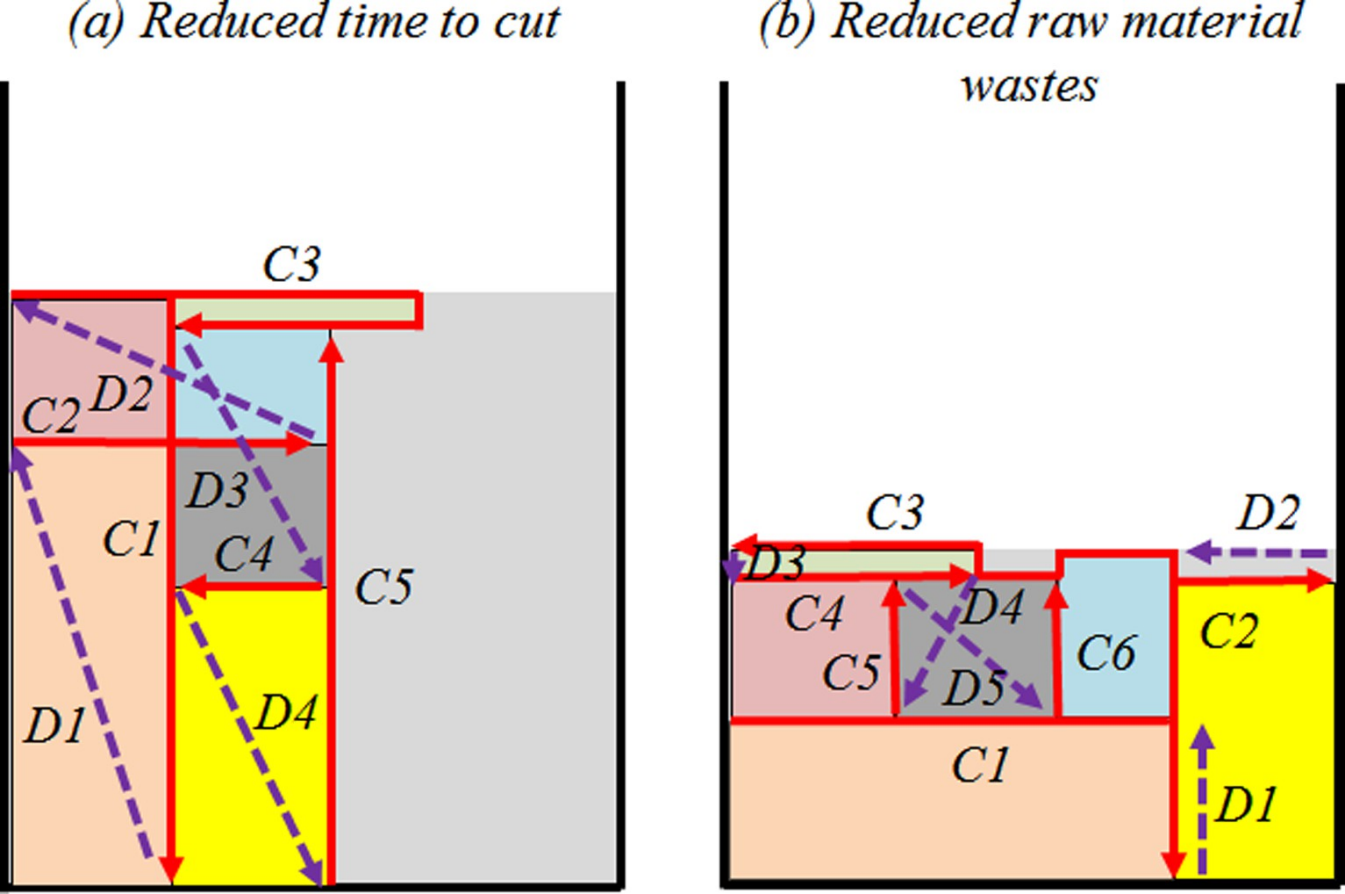
Example of packing layouts with reduced operational cutting processing time (a) and raw material wastes (b).

For the third comparison topic, a tradeoff between reducing the raw material waste and the “Time” required to machine torch “Cut” movements for the plasma sheet metal cutting process is proposed. Fig 7 shows two examples of packing layouts for the same set of rectangles and strip. In Fig 7A, the operational cutting processing time required to “Cut” movements was prioritized using, mainly straight cuts (*C1*, *C2*, *C4*, and *C5*), regardless of the “Displace” movements are long, given the ratio speed difference between “Cut” and “Displace”. Fig 7B shows a minimized raw material waste packing layout, regardless of the number of non-straight cuts and the “Displace” movement behavior.

In the results comparison, for three problem instances (*pt14_24_89b*, *pt7_23_100*, and *N1*), the parameters “Time” and “Cut”, using the optimal solution packing layout, are simultaneously lower compared to alternative solutions, where finding packing layouts with a tradeoff able to reduce simultaneously raw material waste and “Cut” movements, using only the strip height minimization objective function, is hard. The financial cost can aggregate both physical raw material waste and cutting machining gun movement in the same measure. Table 5 shows the total financial cost results based on the raw material waste cost and “Cut” movements given by the active plasma arc value, considering a single sheet metal processing.

The active plasma arc cost is calculated considering the number of inputs consumed as electrical energy (based on required energy power), gas consumed, consumables (replaceable equipped to the machining gun), and manpower (operators). The characteristics and quantity of inputs consumed are based on the operation manual of the Powermax45 XP plasma machine cutting [15]. The raw material waste cost is based on the area of sheet metal not used to allocate rectangles, given by the calculated height $H_{p}^{pi}$ obtained with the solutions packing layout and by the type of sheet metal material, the AISI 1020 mild carbon steel.

A substantial reduction in the machining torch “Cut” movements was not verified for almost all optimal solutions. However, the natural smaller quantity of raw material waste concerning packing layout from alternative solutions partially compensates for excessive active arc movement costs. For the industrial context, the economical impact of both raw material and active arc time waste in the total financial cost is verified when tens or hundreds of sheet metal are cut daily in serial scalable manufacturing planning, where similar small rectangles must be produced with reduced operational cutting processing times, as verified for the automotive sector. For example, to cut 23 rectangles as required for problem instance *pt16_26_84*, the total financial cost to manufacture 50 sheets, considering a daily industrial workday, is approximately $300.00, only considering the costs with raw material and active plasma arc time waste.

## Conclusion

The objective of this research was to compare the machining torch movement behavior using optimal and alternative solutions for rectangular plasma sheet metal cuts. An exploratory analysis was conducted and organized in topics related to the results obtained for 15 problem instances. The possibility of different cut configurations impacting the machining torch movement behavior was the first comparison analysis topic, where a straight cut configuration allows cut movements with the shortest distances, which can be considered the most appropriate to reduce the “Time” required to complete all cutting process. However, the non-straight movements are most common, given the irregular manner in which the rectangles are usually packed in the strip.

In the second comparison topic analyzed, considering all problem instances tested, a direct relation between the comparison parameters, machining torch “Displacement” and “Cut” movement behavior, was not verified, based on reference variables such as speed, the operational cutting processing time required to develop all machining torch movements and operational costs. The third comparison topic, for the total financial cost to proceed with a single sheet metal cutting process using an active plasma arc, physical wastes from raw material usage are predominant compared to wastes obtained with the machining torch movement behavior.

Furthermore, for the main contribution (1), the research framework format systematize the steps and activities developed to obtain two results reports as outputs. Although the methodological research framework usage has been focused on the rectangular 2D-SPP, the simulation conditions and the comparative analysis can be adapted to be used in similar cutting and packing problems’ research. In the main contribution (2), the comparison results show that the packing layout given by alternative solutions can reduce the operational cutting processing time and the distance covered by the machining torch movement during the plasma sheet metal cutting process compared to packing layouts derivate from optimal solutions. As described in all three-comparison topics analyzed, the packing layout trends to be compacted when optimal solutions are considered, in which straight cuts are not privileged to minimize the strip height. As a consequence, the operational cutting processing time is reduced when compared with packing layouts from alternative solutions in which straight cuts are common.

For the main contribution (3), the present study showed not only a scientific contribution to operational research but also a new contribution to improve manufacturing efficiency in the metal-mechanical sector, in particular for the plasma sheet metal cutting process. From an economic perspective, the impact of both raw material and operational cutting processing time wastes from different packing layouts are useful to verify that optimal solutions are more economical options for cutting rectangles when compared to alternative solutions. However, wastes from machining torch movement must be considered to reduce operational costs, especially in the industrial manufacturing context, as verified for the production of small rectangles of steel and aluminum welded to assemble trucks’ bodywork.

The present research deals with different analytic metrics to evaluate solutions, helping to bridge a gap between academic research and effective rectangular 2D-SPP industrial applications. A possibility to simultaneously minimize both the machining torch movement and the strip height to pack all rectangles can be a mathematical model using a multi-objective function concept [47, 48]. Thus, optimal solutions with an ideal packing layout to satisfactorily reach the tradeoff between strip height and torch movement minimization can be found. The optimal solution considering only the strip height minimization as an objective function could not be appropriated to reduce cost in sheet metal cutting process.

As research limitations, only 15 problem instances were selected, given the time required to proceed with all simulation steps for each problem instance, being the number of problem instances was sufficient to perform the comparative analysis and an exploratory discussion divided into three topics, as shown in Section 3. The lack of scientific literature related to the theme presented in this study is also a limitation, due to the impossibility of a parallel between the results and the exploratory analysis developed with other findings from the literature. As for the operational simulation process, the lack of an option to control the “Displace” movement speed is a factor to be improved in the next Mach3® simulation software versions. Finally, the cutting rectangles’ distance of the operational parameters is equal to zero and border cuts were not considered, which generally is not verified for practical sheet metal cutting processes cases where plasma machining is used. Only a single cut is required to separate two adjacent rectangles, being the “Cut” efficient, but with real chances of loss in finishing quality, given the highly active plasma arc heat, which can over-melt the sheet metal surface.

For future studies, our recommendation is to consider the rectangles’ cutting distance equal to or greater than the diameter of the machining torch to avoid rework or loss of cut rectangles. Another factor to be considered is how the plasma machining maintenance operationalization is developed and the difficulty to supply frequently all necessary inputs, which are factors not considered in operational costs studies, despite being significantly impactful in the industrial context.

## References

[1] Ma H, Liu CC. Two-dimensional profile-packing approach for multi-torch flame cutting. International Journal of Production Research. 2007;45(12):2841–2857. doi: 10.1080/00207540600693515

[2] Delmas MA, Pekovic S. Resource Efficiency Strategies and Market Conditions. Long Range Planning. 2015;48(2):80–94. doi: 10.1016/j.lrp.2013.08.014

[3] Wäscher G, Haußner H, Schumann H. An Improved Typology of Cutting and Packing Problems. European Journal of Operational Research. 2007;183(3):1109–1130. doi: 10.1016/j.ejor.2005.12.047

[4] Bennell JA, Oliveira JF, Wäscher G. Cutting and packing. International Journal of Production Economics. 2013;145(2):449–450. doi: 10.1016/j.ijpe.2013.06.021

[5] Neuenfeldt JA, Siluk J, Francescatto M, Stieler G, Disconzi D. A framework to select heuristics for the rectangular two-dimensional strip packing problem. Expert Systems with Applications. 2023;213(C)119202. doi: 10.1016/j.eswa.2022.119202

[6] Neuenfeldt JA, Silva E, Francescatto M, Rosa CB, Siluk J. The rectangular two-dimensional strip packing problem real-life practical constraints: A bibliometric overview. Computers and Operations Research. 2022;137(1)105521:1-19. doi: 10.1016/j.cor.2021.105521

[7] Burke EK, Kendall G, Whitwell G. A New Placement Heuristic for the Orthogonal Stock-Cutting Problem. Operations Research. 2004;52(4):655–672. doi: 10.1287/opre.1040.0109

[8] Lesh N, Marks J, McMahon A, Mitzenmacher M. New Heuristic and Interactive Approaches to 2D Rectangular Strip Packing. ACM Journal of Experimental Algorithmics. 2005;10(1). doi: 10.1145/1064546.1083322

[9] Bortfeldt A. A Genetic Algorithm for the Two-Dimensional Strip Packing Problem with Rectangular Pieces. European Journal of Operational Research. 2006;172(3):814–837. doi: 10.1016/j.ejor.2004.11.016

[10] de Queiroz TA, Miyazawa FK. Two-dimensional strip packing problem with load balancing, load bearing and multi-drop constraints. International Journal of Production Economics. 2013;145(2):511–530. doi: 10.1016/j.ijpe.2013.04.032

[11] Zhang DF, Wei LJ, Leung SCH, Chen QS. A Binary Search Heuristic Algorithm Based on Randomized Local Search for the Rectangular Strip-Packing Problem. INFORMS Journal on Computing. 2013;25(2):332–345. doi: 10.1287/ijoc.1120.0505

[12] Martello S, Monaci M, Vigo D. An Exact Approach to the Strip-Packing Problem. INFORMS Journal on Computing. 2003;15(3):310–319. doi: 10.1287/ijoc.15.3.310.16082

[13] Kenmochi M, Imamichi T, Nonobe K, Yagiura M, Nagamochi H. Exact Algorithms for the Two-Dimensional Strip Packing Problem with and without Rotations. European Journal of Operational Research. 2009;198(1):73–83. doi: 10.1016/j.ejor.2008.08.020

[14] Wang L, Yin AH. A Quasi-Human Algorithm for the Two Dimensional Rectangular Strip Packing Problem: In Memory of Prof. Wenqi Huang. Journal of Combinatorial Optimization. 2016;32(2):416–444. doi: 10.1007/s10878-015-9961-z

[15] Hyperterm. Powermax45 XP® operator manual. Hypertherm®; 2018. Available from: https://www.hypertherm.com/Download?fileId=HYP109170&zip=False.

[16] Radziemski LJ, Cremers AD. Laser Induced Plasmas and Applications. 1st ed. CRC Press; 1990.

[17] Sanda M, Coteana M, Munteanu A. Experimental results concerning the Variation of Surfaxe Roughness parameter (Ra) at Plasma arc cutting of a stainless steel workpiece. International Journal of Modern Manufacturing Technologies. 2010;2(1):2067–3604.

[18] Neuenfeldt Júnior A, Silva E, Miguel Gomes A, Oliveira JF. The Two-Dimensional Strip Packing Problem: What Matters?. Proceedings of the 18th Congress of APDIO, the Portuguese Association of Operational Research. Porto, Portugal. Springer; 2018: 151–164. doi: 10.1007/978-3-319-71583-4_11

[19] Oliveira JF, Neuenfeldt A, Silva E, Carravilla MA. A Survey on heuristics for the Two-Dimensional Rectangular Strip Packing Problem. Pesquisa Operacional. 2016;36(2): 197–226. doi: 10.1590/0101-7438.2016.036.02.0197

[20] Neuenfeldt AJ, Francescatto M, Stieler G, Disconzi D. A Multi-label Transformation Framework for the Rectangular 2D Strip-Packing Problem. Management and Production Engineering Review. 2021;12(4):27–37. doi: 0.24425/mper.2021.139992

[21] Vasilyev I, Ushakov A, Zhang D, Ren J. Generalized multiple strip packing problem: Formulations, applications, and solution algorithms. Computers & Industrial Engineering. 2023;178(1)109096:1–19. doi: 10.1016/j.cie.2023.109096

[22] Fang J, Rao Y, Shi M. A deep reinforcement learning algorithm for the rectangular strip packing problem. PLoS ONE. 2023;18(3)e0282598:1-20. doi: 10.1371/journal.pone.0282598 36928505 PMC10019708

[23] Grandcolas S, Pain-Barre C. A hybrid metaheuristic for the two-dimensional strip packing problem. Annals of Operations Research. 2022;309(1):79–102. doi: 10.1007/s10479-021-04226-6

[24] Cid-Garcia N, Rios-Solis Y. Exact solutions for the 2d-strip packing problem using the positions-and-covering methodology. PLoS ONE. 2021;16(1)e0245267:1–20. doi: 10.1371/journal.pone.0245267 33444394 PMC7808675

[25] Rakotonirainy R, van Vuuren J. Improved metaheuristics for the two-dimensional strip packing problem. Applied Soft Computing Journal. 2020;92(1)106268:1–13.

[26] Alvarez-Valdes R, Parreno F, Tamarit JM. A Branch and Bound Algorithm for the Strip Packing Problem. OR Spectrum. 2009;31(2):431–459. doi: 10.1007/s00291-008-0128-5

[27] Arahori Y, Imamichi T, Nagamochi H. An Exact Strip Packing Algorithm Based on Canonical Forms. Computers & Operations Research. 2012;39(12):2991–3011. doi: 10.1016/j.cor.2012.03.003

[28] Chazelle B. The Bottom-Left Bin-Packing Heuristic: An Efficient Implementation. IEEE Transactions on Computers. 1983;C–32(8):697–707. doi: 10.1109/TC.1983.1676307

[29] Alvarez-Valdes R, Parreno F, and Tamarit JM. A GRASP Algorithm for Constrained Two-Dimensional Non-Guillotine Cutting Problems. Journal of the Operational Research Society. 2005;56(4):414–425. doi: 10.1057/palgrave.jors.2601829

[30] Glover F. Tabu Search: A Tutorial. INFORMS Journal on Applied Analytics. 1990;20(4):74–94. doi: 10.1287/inte.20.4.74

[31] Nemchinsky VA. Plasma flow in a nozzle during plasma arc cutting. Journal of Physics D: Applied Physics. 1998;31(21). doi: 10.1088/0022-3727/31/21/016

[32] Nemchinsky VA, Severance WS. What We Know and What We Do Not Know about Plasma Arc Cutting. Journal of Physics D: Applied Physics. 2006;39(22):423–438. doi: 10.1088/0022-3727/39/22/R01

[33] Chen JC, Li y, Cox RA. Taguchi-Based Six Sigma Approach to Optimize Plasma Cutting Process: An Industrial Case Study. International Journal of Advanced Manufacturing Technology. 2009;41(7–8):760–769. doi: 10.1007/s00170-008-1526-1

[34] Salonitis K, Vatousianos S. Experimental Investigation of the Plasma Arc Cutting Process. Proceeding of the 45th CIRP Conference on Manufacturing Systems; 2012. United Kingdom. Elsevier; 2012:287–292. doi: 10.1016/j.procir.2012.07.050

[35] Deli J, Bo Y. An Intelligent Control Strategy for Plasma Arc Cutting Technology. Journal of Manufacturing Processes. 2011;13(1):1–7. doi: 10.1016/j.jmapro.2010.08.003

[36] International Organization for Standardization. Thermal cutting ‐ Classification of thermal cuts ‐ Geometrical product specification and quality tolerances (ISO Standard No. 9013:2002); 2002. Available from: https://www.iso.org/standard/29998.html.

[37] Wang PY, Valenzela CL. Data Set Generation for Rectangular Placement Problems. European Journal of Operational Research. 2001;134(2):378–391. doi: 10.1016/S0377-2217(00)00263-0

[38] Hopper E, Turton BCH. Empirical Investigation of Meta-Heuristic and Heuristic Algorithms for a 2D Packing Problem. European Journal of Operational Research. 2001;128(1):34–57. doi: 10.1016/S0377-2217(99)00357-4

[39] Ferreira EP, Oliveira FJ. A note on Fekete and Schepers’ algorithm for the non-guillotinable two-dimensional packing problem. INESC Porto; 2005.

[40] Beasley JE. Algorithms for Unconstrained Two-Dimensional Guillotine Cutting. Journal of the Operational Research Society. 1985a;36(4):297–306. doi: 10.1057/jors.1985.51

[41] Christofides N, Whitlock C. An algorithm for two-dimensional cutting problems. Operations Research. 1977;25(1):30–44. doi: 10.1287/opre.25.1.30

[42] Beasley JE. Exact two-dimensional non-guillotine cutting tree search procedure. Operations Research. 1985b;33(1):49–64. doi: 10.1287/opre.33.1.49

[43] Berkey JO, Wang PY. Two-Dimensional Finite Bin-Packing Algorithms. Journal of the Operational Research Society. 1987;38(5):423–429. doi: 10.1057/jors.1987.70

[44] Martello S, Vigo D. Exact Solution of the Two-Dimensional Finite Bin Packing Problem. Management Science. 1998;44(3):388–399. doi: 10.1287/mnsc.44.3.388

[45] Bengtsson B-E. Packing Rectangular Pieces ‐ a Heuristic Approach. Computer Journal. 1982;25(3):353–357. doi: 10.1093/comjnl/25.3.353

[46] Neuenfeldt A, Silva E, Gomes M, Soares C, Oliveira JF. Data Mining Based Framework to Assess Solution Quality for the Rectangular 2D Strip-Packing Problem. Expert Systems with Applications. 2019;118:365–380. doi: 10.1016/j.eswa.2018.10.006

[47] de Armas J, Leon C, Miranda G, Segura C. Optimisation of a multi-objective two-dimensional strip packing problem based on evolutionary algorithms. International Journal of Production Research. 2010;48(7):2011–2028. doi: 10.1080/00207540902729926

[48] Liu Y, Chu C, Yu Y. Aggregated state dynamic programming for a multiobjective two-dimensional bin packing problem. International Journal of Production Research. 2012;50(15):4316–4325. doi: 10.1080/00207543.2011.622309

